# Depression Improvement Correlates With Lower TMS Intensity in a Randomized Trial With Real‐Time E‐Field Modeling

**DOI:** 10.1002/hbm.70607

**Published:** 2026-08-04

**Authors:** Prem Ganesh, Hakjoo Kim, Jamie Kweon, Mark A. Halko, Kevin A. Caulfield, Joshua C. Brown

**Affiliations:** ^1^ Brain Stimulation Mechanisms Laboratory, Division of Depression and Anxiety Disorders McLean Hospital Belmont Massachusetts USA; ^2^ Department of Psychiatry Harvard Medical School Boston Massachusetts USA; ^3^ Brain Stimulation Division, Department of Psychiatry and Behavioral Science Medical University of South Carolina Charleston South Carolina USA

**Keywords:** computational modeling, E‐field, parameter space, TMS

## Abstract

Current transcranial magnetic stimulation (TMS) practice uses fixed‐percentage motor threshold dosing, conventionally 120% rMT, for depression treatment. Individual anatomical variability may result in substantially different cortical electric‐Field (E‐Field) strengths across patients. We used prospective real‐time E‐Field modeling to characterize individual TMS intensity requirements and examine associations with clinical outcomes. Twenty‐eight subjects with major depressive disorder received single‐day accelerated intermittent theta‐burst stimulation (10 sessions, 1800 pulses/session) with real‐time E‐Field‐guided dosing targeting M1‐equivalent stimulation at left dorsolateral prefrontal cortex (DLPFC). Required %rMT for motor‐equivalent DLPFC E‐Field ranged from 49.7%–150.4% rMT (mean = 99.7% ± 18.9%), with 53.6% of subjects requiring less than 100% rMT. Real‐time E‐Field‐guided dosing achieved 48.1% better precision than conventional 120% rMT in approximating motor‐equivalent E‐Field delivery (*t*(27) = 2.45, *p* = 0.021). Three dosing frameworks were tested against change in QIDS‐SR16 scores: delivered %rMT was not significantly associated with outcomes (*ρ* = −0.15, *p* = 0.436), while both the ratio of DLPFC to M1 E‐Field strength (*ρ* = −0.49, *p* = 0.008) and absolute DLPFC E‐Field strength (*ρ* = −0.49, *p* = 0.008) were significantly negatively correlated with greater symptom reduction. These findings demonstrate substantial interindividual variability in cortical E‐Field delivery under fixed‐percentage dosing, and that real‐time E‐Field guidance more precisely approximates motor‐equivalent stimulation than conventional 120% rMT. The association of lower absolute DLPFC E‐Field strength with greater symptom reduction challenges the assumption that higher stimulation intensity produces better clinical outcomes, and warrants systematic investigation in larger trials.

## Key Points

− Individual TMS dosing requirements vary substantially (50%–150% rMT) to achieve M1‐equivalent E‐Field strength at DLPFC.
− Highly variable %rMT was not associated with improved clinical outcomes, while both lower DLPFC‐to‐M1 E‐Field ratio and lower absolute DLPFC E‐Field strength were significantly associated with greater symptom reduction.
− Lower absolute DLPFC E‐Field strength was associated with greater clinical improvement, challenging the assumption that higher stimulation intensity produces better outcomes.

## Graphical abstract

We used E‐Field modeling to compare two TMS dosing paradigms for treating depression at the left DLPFC: real‐time E‐Field guidance (motor‐equivalent targeting) versus conventional 120% motor threshold. Lower DLPFC E‐Field strengths were associated with better clinical outcomes.

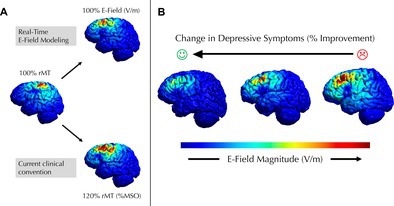

## 1 Introduction

Transcranial magnetic stimulation (TMS) is an FDA‐approved treatment for major depressive disorder; however, clinical response rates remain modest and variable, ranging from 40% to 60% across trials and naturalistic studies (Carpenter et al. 2012; Cirillo et al. 2019; Conelea et al. 2017; George et al. 2010; O'Reardon et al. 2007). Multiple factors potentially contribute to this variability, including target selection, stimulation protocol parameters, and patient characteristics (Caulfield and Brown 2022; Cerins et al. 2024; Fox et al. 2012; Hutton et al. 2023). Among these, stimulation intensity represents a readily modifiable parameter that has received limited systematic investigation despite its potential influence on treatment outcomes.

Current clinical practice uses resting motor threshold (rMT), a visible indicator of neuronal activation, as a proxy for determining stimulation intensity at non‐motor brain targets. Following rMT determination at the primary motor cortex (M1), a fixed percentage, conventionally 120% of rMT, is applied to the left dorsolateral prefrontal cortex (DLPFC) for depression treatment (Addicott et al. 2024; Rossi et al. 2021; Rothwell et al. 1999). This approach assumes that distance from coil‐to‐cortex and excitability requirements for M1 activation translate to the DLPFC in a fixed ratio for each patient.

Computational modeling of TMS‐induced electric‐Fields (E‐Fields) has emerged as a tool to estimate the actual cortical stimulation delivered to brain targets, accounting for individual anatomical differences in skull geometry, brain morphology, and tissue conductivity (Dannhauer et al. 2024; Quinn et al. 2023; Van Hoornweder et al. 2023). Offline approaches using finite element method (FEM) modeling have demonstrated substantial interindividual variability in required intensities when targeting equivalent E‐Field strengths (Dannhauer et al. 2024; Van Hoornweder et al. 2023; Caulfield et al. 2021). Two notable studies sought to determine the exact translation between M1 and DLPFC as measured by induced E‐Field. Nahas and colleagues reported that subjects required, on average, 114% of rMT to compensate for differences in coil‐to‐cortex distance at the DLPFC, while Caulfield and colleagues found a mean of 134% rMT was needed for equivalent E‐Field strength at the DLPFC relative to M1. Both substantially varied from the 120% convention for many individuals (Caulfield et al. 2021; Nahas et al. 2004). These findings suggest that fixed‐percentage dosing may inadequately account for individual anatomical variation and could over or under‐stimulate, and in turn, yield suboptimal outcomes.

Recent technological advances enable real‐time E‐Field estimation during TMS delivery using spherical head models integrated into neuronavigation systems (Daneshzand et al. 2021; Nummenmaa et al. 2013; Ruohonen and Karhu 2010). While less anatomically detailed than offline FEM approaches, which require hours of computation, real‐time spherical modeling provides immediate feedback on estimated cortical E‐Field strength during treatment planning. This enables prospective individualized intensity adjustments based on estimated cortical dose rather than relying solely on fixed rMT percentages.

Despite these advances, fundamental questions remain unresolved regarding optimal TMS intensity for treating depression. The 120% rMT convention lacks clear empirical foundation from systematic testing; the accuracy of real‐time spherical models compared to gold‐standard FEM has not been validated in prospective clinical implementation; and the relationship between delivered cortical E‐Field strength and clinical outcomes for depression has not been systematically examined. In this study, we prospectively implemented real‐time E‐Field‐guided dosing in 28 subjects with major depressive disorder receiving single‐day accelerated intermittent theta‐burst stimulation (iTBS). In this report, we characterized individual variability in intensity requirements for achieving M1‐equivalent E‐Field strength at the DLPFC target, compared real‐time spherical head modeling against gold‐standard FEM simulations, and tested clinical outcomes against three stimulation intensity dosing models: (1) conventional 120% rMT; (2) motor‐equivalent E‐Field at DLPFC (matching the V/m obtained from MT); (3) absolute E‐Field strength. By linking individualized E‐Field dosing to both biophysical modeling and clinical outcomes, this study aims to establish an empirical foundation for personalized TMS intensity selection.

## 2 Methods

### 2.1 Study Overview

The data used in this secondary analysis were obtained from a McLean Hospital IRB‐approved, randomized, double‐blind, and placebo‐controlled trial enrolling 30 participants with MDD. Subjects were randomized to receive either 250 mg d‐cycloserine or placebo capsules (McLean Hospital Pharmacy). All 30 subjects (17 women, mean age = 38.5, standard deviation (SD) = 16.0, range = 18–69) underwent magnetic resonance imaging (MRI) and completed the single‐day accelerated‐TMS protocol as outlined below. MRI data were acquired on a 3T Siemens Prisma scanner (Siemens Healthcare, Erlangen, Germany) located at the McLean Imaging Center. The imaging protocol included a high‐resolution T1‐weighted MPRAGE anatomical MRI scan for each subject (TR = 2.3 s, TE = 2.32 ms, TI = 0.9 s, flip angle = 8°, 0.9 mm isotropic voxels, 256x256 matrix, acceleration factor = 2). Each T1‐weighted anatomical scan was uploaded to Nexstim neuronavigation software (Nexstim Plc, Helsinki, Finland).

Using Nexstim NBR 1.0 system (Nexstim Plc, Helsinki, Finland), single‐pulse TMS was first applied over the M1 while simultaneous EMG recording of the contralateral right abductor pollicis brevis (APB) muscle to determine rMT (using Nexstim's automated estimation algorithm) for each subject. All participants received 10 sessions of 1800‐pulse intermittent theta‐burst stimulation (iTBS) (50‐Hz triplet bursts, 5‐Hz carrier frequency) spaced at least 50 min, as previously described (Huang et al. 2005; Cole, Phillips, et al. 2022). The individualized left DLPFC cortical target was derived from structural anatomy (Mylius et al. 2013). The coil orientation was individualized to be approximately 90 degrees perpendicular to the target gyrus in the DLPFC. In addition to MEP and TMS‐evoked potential data, which will be reported separately, the 16‐item Quick Inventory of Depressive Symptomatology–Self Report (QIDS‐SR16) (Rush et al. 2003) was collected twice: at baseline prior to iTBS administration and 1 week following the accelerated iTBS protocol.

### 2.2 TMS Dosing

The Nexstim NBR 1.0 performs real‐time monitoring of the maximum electric field strength (*E*_max_) at the targeted cortical depth and chosen percentage of maximum stimulator output (%MSO) based on their spherical head model E‐Field modeling algorithm (Ruohonen and Karhu 2010). Each subject underwent cortical mapping of the M1. rMT was then determined at M1 using the Nexstim MT‐curve method (Julkunen et al. 2011). The corresponding maximum *E*_max_ at the chosen cortical depth was recorded. The coil was then repositioned to the left DLPFC target, maintaining the same cortical depth. The %MSO was adjusted to match the *E*_max_ recorded during rMT determination. This personalized dosing procedure was performed immediately prior to the first treatment session. The determined dose for the DLPFC target remained constant for all 10 treatments.

### 2.3 MRI Preprocessing and Segmentation

**FIGURE 1:**
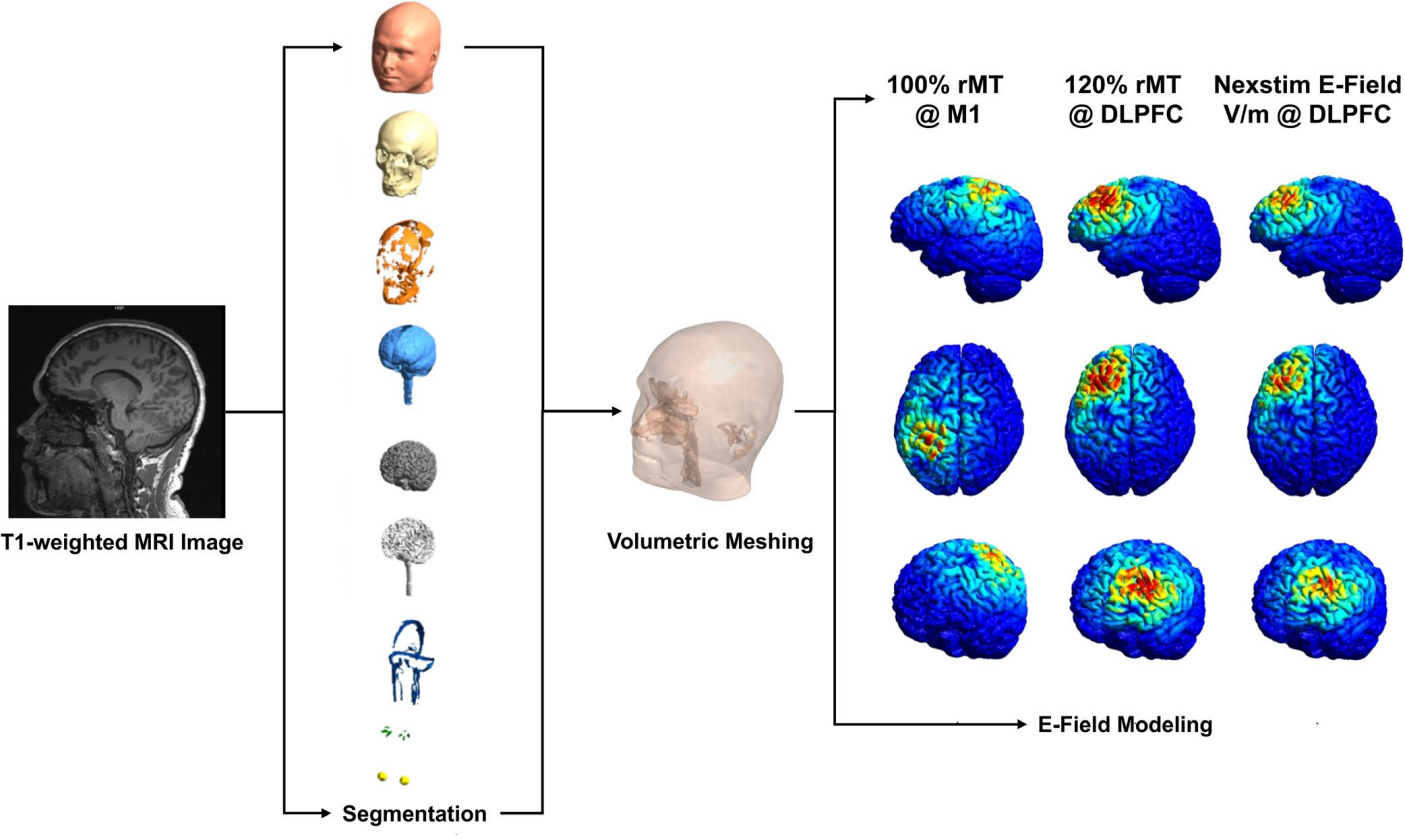
E‐Field modeling flowchart in a representative participant. Segmentation depicts all 9 tissue layers to scale (in order: skin, compact bone, spongy bone, CSF, gray matter, white matter, blood, muscle, eyeballs). All images shown are an example taken from one subject.

Cortical reconstruction and volumetric segmentation were first performed on T1w magnetic resonance (MR) images with Freesurfer image analysis suite, which is documented and freely available online (http://surfer.nmr.mgh.harvard.edu/). Technical details of this pipeline were described in detail previously (Fischl et al. 2002). MR images and Freesurfer segmentations were then processed and segmented using the Computational Anatomy Toolbox (CAT12) implemented within the Statistical Parametric Mapping (SPM12) software using the charm segmentation pipeline (Puonti et al. 2020). Following preprocessing, voxel‐wise tissue classification segmented each MR image into skin, spongy bone, compact bone, CSF, blood, muscle, eyeballs, white matter, and gray matter (Puonti et al. 2020; Nielsen et al. 2018). Tissue layers were then combined into a 3D volumetric mesh for modeling (Figure 1). Two subjects were omitted due to poor MRI quality (excessive motion/artifacts prevented reliable tissue segmentation), resulting in a final sample of 28 subjects. Demographic information can be found in Table 1.

**TABLE 1:** Demographic information for all subjects (*n* = 28).

Demographics	Total	Placebo	d‐cycloserine
*N*	28	13	15
Age	38.5 ± 16.0	41.8 ± 15.4	35.2 ± 16.4
Sex (*n*, % F)	17, 60.7%	10, 76.9%	7, 46.7%
Race			
White (*n*, %)	27, 96.4%	13, 100%	14, 93.3%
Asian (*n*, %)	1, 3.6%	0, 0%	1, 6.67%
Black/African American (*n*, %)	0, 0%	0, 0%	0, 0%
Clinical outcomes—QIDS‐SR16			
Baseline	14.0 ± 0.9	14.9 ± 1.5	13.1 ± 1.1
1‐Week post‐TMS	11.3 ± 1.0	11.8 ± 1.9	10.9 ± 1.1
Percent improvement (%)	16.0% ± 7.6%	22.4% ± 8.0%	10.5% ± 12.5%
Response	21.4%	23.1%	20.0%
Remission	17.9%	23.1%	13.3%

### 2.4 Coil Modeling

A custom computational model of the Nexstim Cooled Coil was developed based on a figure‐of‐eight winding pattern. The coil consists of two circular loops of wire arranged symmetrically, generating a combined E‐Field at their intersection. Dimensions of the coil windings were approximated based on physical measurements of both the outer casing and from x‐ray images. The coil wire path was generated computationally to simulate the actual conductor arrangement, including the input and output leads. The rate of change of current in the coil (*dI/dt*) was obtained under agreement from the manufacturer (Nexstim Plc, Helsinki, Finland).

A custom Python (version 3.11) script, adapted from the SimNIBS TMS coil simulation framework, was used to implement the coil model. The coil windings were modeled as a parametric spiral, following the geometry of an idealized figure‐of‐eight coil (Equation 1), where *r(ϕ)* is the radius at angle *ϕ, D*_outer_ is the outer spiral diameter, and *ϕ* is the angle along the spiral ranging from *π/6* to *ϕ*_max_. Each loop was discretized into 1000 elements for high‐accuracy field calculations. The complete TMS coil was exported for use in SimNIBS simulations.

(1) $$r\left(\phi \right)=\frac{D_{\text{outer}}}{2}-\frac{d_{\text{wire}}}{2}\frac{\phi }{2\pi }$$

### 2.5 FEM E‐Field Modeling

Three TMS simulations were computed for each subject using SimNIBS 4.1.0 software and custom MATLAB scripts (Saturnino et al. 2019): 100% rMT at M1, 120% rMT at DLPFC, and individualized E‐Field dose at DLPFC (Figure 1). Coil positions and orientations with coordinates were extracted from the Nexstim neuronavigation software.

To calculate individual intensity requirements for motor‐equivalent DLPFC stimulation, the E‐Field was extracted from each subject's FEM simulation and normalized to a unit current derivative of 1 A/s. The MSO required to achieve M1‐equivalent E‐Field strength at the DLPFC target was then calculated using Equation (2), where $E_{M1}$ is the mean E‐Field at M1 at motor threshold, $E\;{\left(\text{scaled}\right)}_{\text{DLPFC}}$ is the normalized DLPFC E‐Field at 1 A/s, and dIdt is the coil specific rate of current change per percent MSO.

(2) MSOoptimal=EM1EscaledDLPFC×dIdt

**FIGURE 2:**
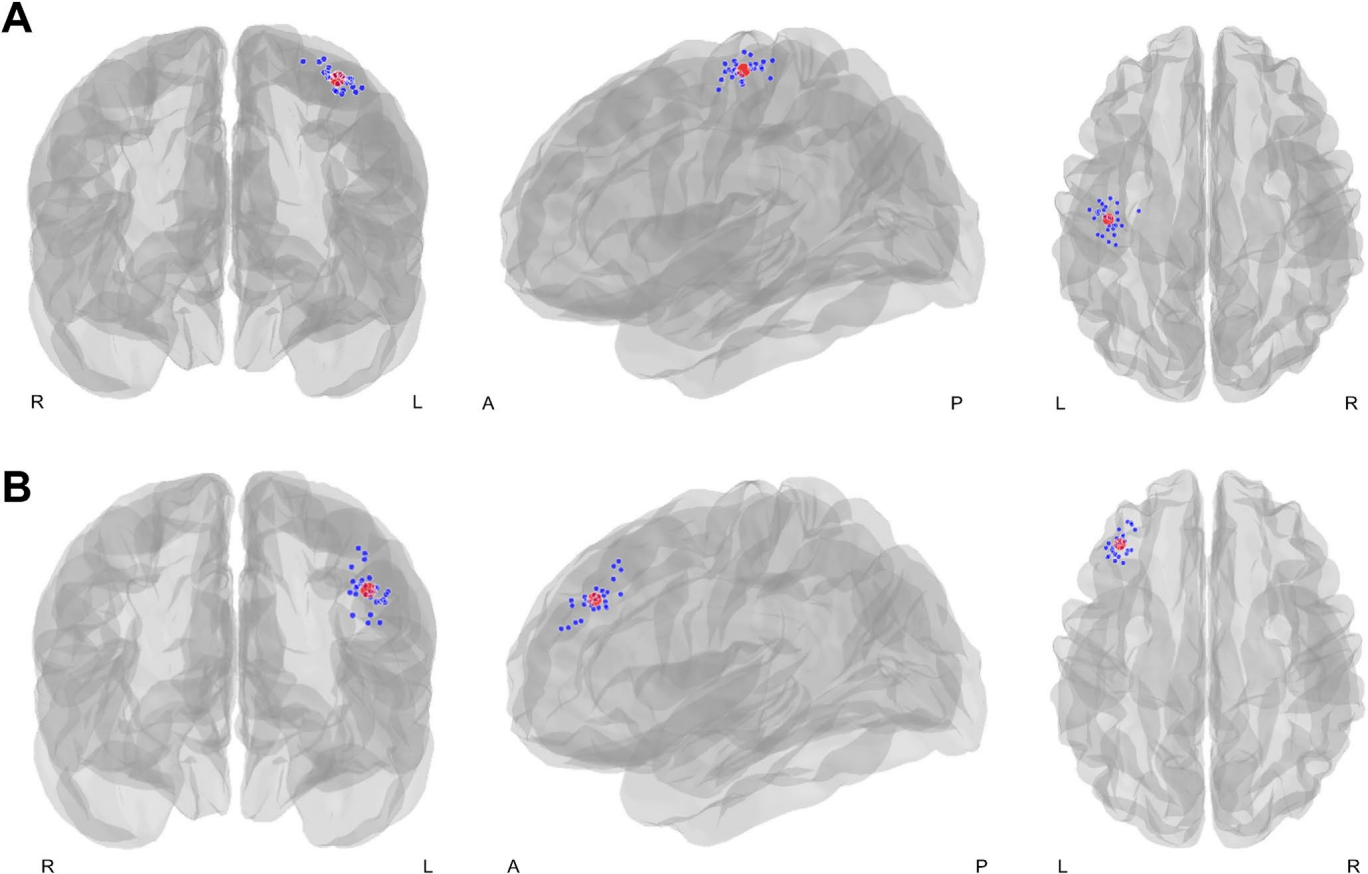
(A) MNI coordinates for the left M1 hotspot as determined by motor mapping prior to motor threshold determination. (B) Individualized left DLPFC target for iTBS as determined by the study physician. Blue = Individual targets, Red = MNI average across all subjects.

We report average Montreal Neurological Institute (MNI) coordinates (Mazziotta et al. 1995) for the left M1 hotspot: (−37, −19, 58) and left DLPFC: (−36, 31, 32) (Figure 2).

E‐Field distribution within the head model was computed for each simulation using SimNIBS FEM solver and with standard conductivity values—White matter: 0.126 S/m, Gray matter: 0.275 S/m, CSF: 1.654 S/m, Compact bone: 0.008 S/m, Spongy bone: 0.025 S/m, Scalp: 0.465 S/m, Eyeballs: 0.5 S/m, Blood: 0.465 S/m, Muscle: 0.16 S/m (Puonti et al. 2020; Gabriel et al. 2009; Opitz et al. 2017; Wagner et al. 2004).

### 2.6 Region of Interest (ROI) Analysis

The spatial distribution of the induced E‐Fields calculated in each of the three simulations was analyzed within a region of interest (ROI) for all subjects following parameters used in prior literature (Van Hoornweder et al. 2023; Caulfield et al. 2021, 2022; Cho et al. 2023; Arias et al. 2025). The ROI is defined as a 10 mm radius sphere centered at the cortical projection directly under the center of the TMS coil at the same depth where rMT and DLPFC target were determined. A gray matter mask was applied at the ROI to ensure only E‐Fields in gray matter tissue contributed to the analysis. E‐Field strength was defined as the volume‐weighted mean E‐Field magnitude within the ROI, consistent with the most commonly reported metric in TMS E‐Field literature (Van Hoornweder et al. 2023). Figure S1 shows E‐Field strength at M1 and DLPFC as modeled by several commonly used metrics including 99th percentile within the ROI, within the whole brain, and the real‐time spherical head model estimate from the Nexstim neuronavigation system to further contextualize the primary outcome within the broader literature (Figure S1). Robustness of the primary 10 mm ROI across 5, 10, and 15 mm radii is shown in Figure S2. Skin‐to‐cortex distance was 12.7 ± 0.3 mm at M1 and 11.3 ± 0.4 mm at DLPFC (t(27) = 3.82, *p* < 0.001, Figure S3). All analyses were performed on subject‐specific mesh files. Subject‐level ROI data including Mean, Maximum, and 99th percentile of E‐Field strength as well as the number of tetrahedra within each ROI are reported in Table S1.

### 2.7 Data Analysis

Descriptive statistics are reported as mean ± standard deviation unless otherwise specified. Normality of distributions was assessed using Shapiro–Wilk tests. rMT and all E‐Field metrics were normally distributed (all *p* > 0.05) and analyzed using Pearson correlations. Clinical improvement (QIDS‐SR16 Reduction) and required %rMT showed non‐normal distributions (*p* < 0.05), and Spearman rank correlations were therefore used for all clinical outcome analyses. Clinical improvement is reported as raw change in QIDS‐SR16 score (pre minus post), where positive values indicate symptom improvement. QIDS‐SR16 scores and changes for each subject are reported in Table S2.

To test theoretical dosing frameworks, we calculated three metrics: (1) delivered %rMT (MSO‐based conventional framework), (2) delivered E‐Field as a percentage of M1 E‐Field at motor threshold (motor equivalence), and (3) absolute DLPFC E‐Field strength. Each framework was tested against change in QIDS‐SR16 using Spearman correlations. d‐cycloserine/placebo group randomization was included as a covariate using partial correlation. For significant correlations, 95% confidence intervals were calculated using bootstrapping with 10,000 paired resampled iterations. Paired *t*‐tests compared dosing precision between real‐time E‐Field‐guided and conventional 120% rMT methods. All clinical analyses were exploratory given the modest sample size (*n* = 28) and are presented with exact *p*‐values. Statistical analyses were performed in Python 3.11 using scipy.stats and statsmodels packages.

## 3 Results

### 3.1 E‐Field Scaling Across Brain Regions

**FIGURE 3:**
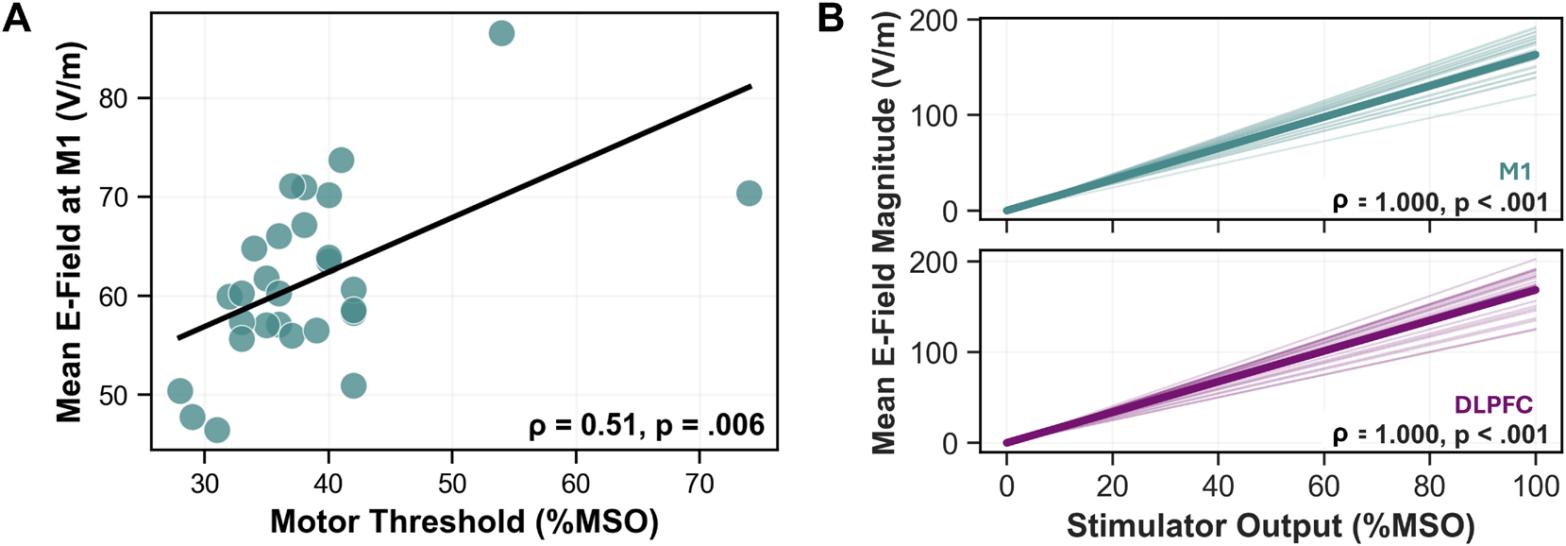
E‐Field scaling across brain regions. (A) Motor threshold versus M1 E‐Field strength at 100% rMT (*ρ* = 0.51, *p* = 0.006). (B) %MSO and E‐Field strength relationships showing individual subjects (light lines, *n* = 28) and group average (bold line, *ρ* = 1.000, *p* < 0.001) at M1 (top) and DLPFC (bottom).

Motor threshold determination across subjects yielded an average of 42.4% ± 7.8% MSO (range: 28.0%–74.0%). Motor threshold correlated significantly with M1 E‐Field strength at 100% rMT (*ρ = 0.51, p* = 0.006; Figure 3A). To characterize individual variability in E‐Field sensitivity, we computed subject‐specific E‐Field scaling factors by normalizing each subject's FEM simulation to a unit current derivative of 1 A/s, resulting in a physics‐based mapping between stimulator output and induced E‐Field magnitude. As expected, group‐averaged E‐Field magnitude scaled linearly with stimulator output at both targets (*ρ* = 1.000, *p* < 0.001; Figure 3B) consistent with the linear scaling properties of FEM models. Individual scaling factors varied substantially across subjects (M1: 1.63 ± 0.22 V/m per %MSO, CV = 13.6%, range = 0.95–1.92; DLPFC: 1.69 ± 0.21 V/m per %MSO, CV = 12.4%, range = 1.25–2.03), suggesting that a fixed %MSO produces meaningfully different cortical E‐Field strengths across individuals. Scaling factors did not differ significantly between M1 and DLPFC targets (*t*(27) = −0.96, *p* = 0.344). Interindividual variability in E‐Field scaling may be consistent across brain regions, rather than target specific.

### 3.2 Dosing Precision and Motor‐Equivalent Intensity Requirements

**FIGURE 4:**
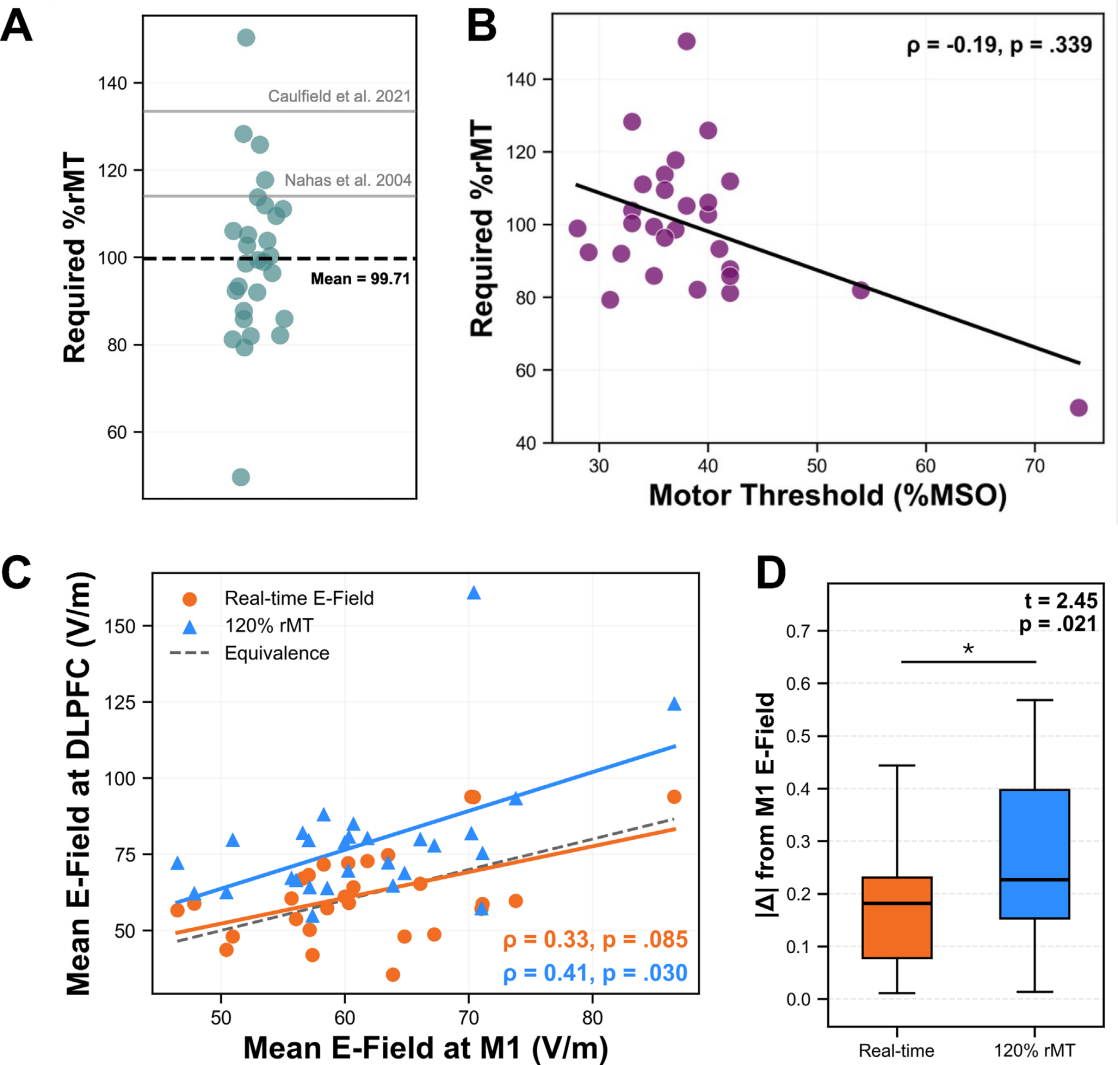
Dosing precision and motor‐equivalent intensity requirements. (A) Distribution of required %rMT to achieve M1‐equivalent DLPFC E‐Field (mean = 99.7% ± 18.9%, range = 49.7%–150.4%). (B) Percentage of machine stimulator output vs. required %rMT (*ρ* = −0.19, *p* = 0.339). (C) M1 vs. DLPFC E‐Field correlations. Orange circles = real‐time method (*ρ* = 0.33, *p* = 0.085); blue triangles = 120% rMT method (*ρ* = 0.41, *p* = 0.030); black dashed line = perfect equivalence. (D) Absolute deviation from M1‐E‐Field. Mean deviation Real‐time E‐Field = 0.170, 120% = 0.293 (*t*(27) = 2.45, *p* = 0.021, Cohen's *d* = 0.46).

Given the established linearity, we calculated the specific %rMT each subject would require to achieve M1‐equivalent E‐Field strength in the DLPFC, directly testing whether a fixed percentage of rMT appropriately doses across individuals. Subjects required a mean of 99.7% ± 18.9% rMT (range: 49.7%–150.4%) to induce DLPFC E‐Fields equivalent to M1 at 100% rMT (Figure 4A); the majority of subjects (53.6%, *n* = 15) required less than 100% rMT, 10 subjects (35.7%) required 100%–120% rMT, and three subjects (10.7%) required greater than 120% rMT. Motor threshold showed a negative correlation with required DLPFC intensity (*ρ* = −0.19, *p* = 0.339; Figure 4B), suggesting that subjects with higher motor thresholds required relatively lower DLPFC intensities to achieve equivalent E‐Field delivery, though this was not significant.

To evaluate dosing precision, we compared real‐time E‐Field‐guided dosing against conventional 120% rMT dosing in achieving M1‐equivalent E‐Fields at the DLPFC target. Both methods showed linear relationships between M1 and DLPFC E‐Fields (real‐time: *ρ* = 0.33, *p* = 0.085, slope = 0.846; 120% rMT: *ρ* = 0.41, *p* = 0.030, slope = 1.276; Figure 4C). However, when evaluating overall accuracy in achieving the target equivalent E‐Field, the real‐time method demonstrated superior performance. Mean absolute deviation from the target ratio of 1.0 was significantly smaller for real‐time dosing (0.170 vs. 0.293, *t*(27) = 2.45, *p* = 0.021, Cohen's *d* = 0.46; Figure 4D), and root mean square error from perfect equivalence was lower for the real‐time method (12.75 V/m vs. 24.55 V/m), representing 48.1% improved precision on average.

### 3.3 Relationship Between Intensity and Clinical Outcomes

Subjects were randomized to receive either placebo or d‐cycloserine. Both groups showed normally distributed QIDS‐SR16 change scores (Placebo: *p* = 0.417; DCS: *p* = 0.466), and a Welch *t*‐test revealed no significant difference in score reduction between groups (Placebo: 2.93 ± 3.77 points; DCS: 1.93 ± 4.55 points; *t* = 0.63, *p* = 0.532, *d* = 0.24); therefore, groups were combined for subsequent analyses. QIDS‐SR16 scores decreased by a mean of 2.43 ± 4.14 points (range: −11 to +8), with five subjects (17.9%) meeting responder criteria (≥ 50% reduction from baseline). To examine whether TMS intensity is associated with clinical outcomes, we tested three dosing frameworks.

**FIGURE 5:**
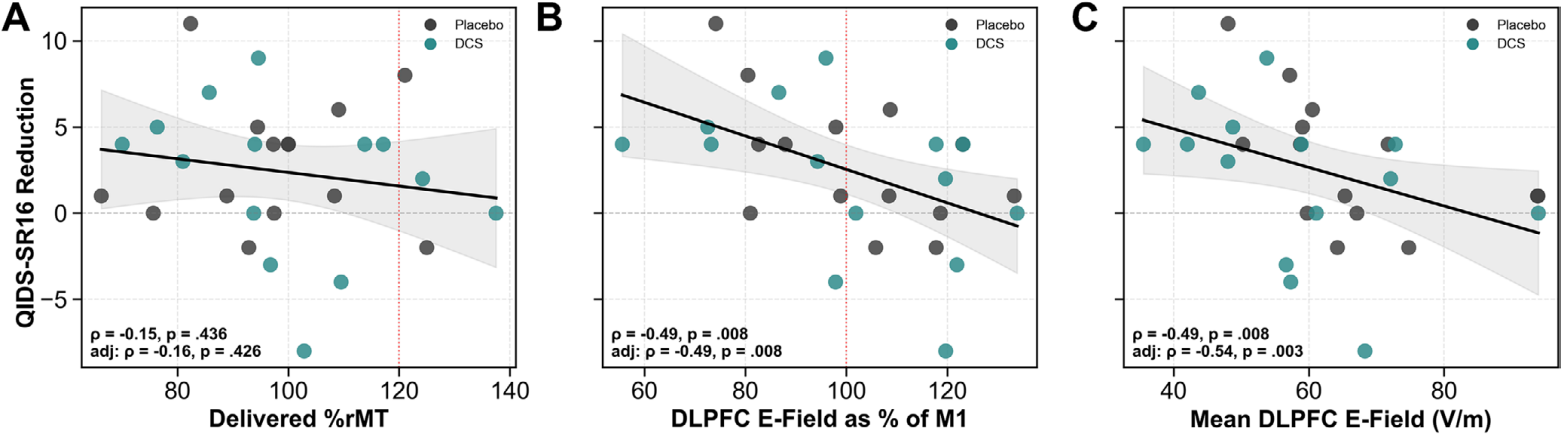
Change in depression scale correlates only with precision intensity metrics. *Y* axis (pre—post) calculation denotes more positive scores (upgoing) as better clinical response. Exploratory clinical correlations. (A) Delivered %rMT versus clinical improvement (*ρ* = −0.15, *p* = 0.436). (B) Percentage of rMT‐equivalent E‐Field versus clinical improvement (*ρ* = −0.49, *p* = 0.008). (C) Absolute DLPFC E‐Field strength versus clinical improvement (*ρ* = −0.49, *p* = 0.008, 95% CI [−0.20, −0.69]).

First, we examined whether the delivered stimulator output as a percentage of motor threshold (%rMT) was associated with clinical outcomes. Clinical protocols for depression stipulate an intensity of 120% of rMT, which is intolerable for a sizeable minority of patients which can leads to concerns that TMS will be less effective. We found no significant relationship between delivered %rMT and change in QIDS‐SR16 scores (*ρ* = −0.15, *p* = 0.436; Figure 5A). Second, we tested whether clinical responses correlated with relative intensity at the DLPFC compared to motor‐equivalent E‐Field strength (i.e., DLPFC/M1 ratio = 100%). We found a significant negative correlation between outcomes and percentage of M1 E‐Field delivered to DLPFC (*ρ* = −0.49, *p* = 0.008; Figure 5b), which remained after adjusting for d‐cycloserine group assignment (adj. *ρ* = −0.49, *p* = 0.008). Third, we examined whether absolute DLPFC E‐Field strength correlated with clinical outcomes. Here, we did see a significant negative correlation with clinical outcomes where weaker absolute intensity as measured by V/m produced better outcomes (*ρ* = −0.49, *p* = 0.008, 95% CI [−0.20, −0.69]; Figure 5C), which was strengthened after adjusting for group assignment (adj. *ρ* = −0.54, *p* = 0.003). While these analyses were performed on Mean E‐Field within the ROIs, we report the same analyses in the supplemental material using 99th percentile within the ROI, which were directionally consistent and significant with these results (Figure S4).

Consistent with an inverse intensity‐outcome relationship, dose–response analysis by intensity quartiles generally showed a decline in QIDS‐SR16 score change with increasing E‐Field intensity: Q1 (lowest) = 5.4 ± 2.8 points, Q2 = 3.3 ± 5.0 points, Q3 = −0.4 ± 4.2 points, Q4 (highest) = 1.4 ± 2.1 points (Kruskal‐Wallis: *H*(3) = 8.21, *p* = 0.042) (Figure S5). Responders (*n* = 5) received mean DLPFC E‐Field of 53.0 ± 7.2 V/m compared to 64.0 ± 15.4 V/m for non‐responders (Mann–Whitney *U* = 31.00, *p* = 0.121). Interaction analyses revealed no significant moderators of drug, age, sex, or baseline severity affected the intensity‐outcome relationship: d‐cycloserine × intensity (*β* = −0.029, *p* = 0.788), baseline severity × intensity (*β* = −0.014, *p* = 0.420), sex × intensity (*β* = −0.135, *p* = 0.209), age × intensity (*β* = −0.004, *p* = 0.311).

## 4 Discussion

In this study, we used prospective E‐Field modeling to dose TMS intensity and examined individual variability in TMS intensity requirements to test whether different intensity‐related metrics are associated with clinical outcomes. We found that achieving motor‐equivalent E‐Field strength in the DLPFC required substantially different intensities across individuals (range: 49.7%–150.4% rMT), with the majority of subjects (53.6%) requiring less than the conventional 120% rMT standard. Real‐time E‐Field‐guided dosing achieved, on average, 48.1% better precision than conventional 120% rMT dosing in delivering target E‐Field strengths.

The 3‐fold variability in required intensities (49.7%–150.4% rMT) demonstrates that fixed‐percentage approaches inadequately account for individual anatomical and physiological differences. This variability substantially exceeds previous reports; Nahas and colleagues found subjects required 103%–141% MT to depth correct from M1 to DLPFC (mean ~ 122%) in 18 subjects (Nahas et al. 2004), while Caulfield and colleagues reported a mean of 134% MT (range not specified) based on retrospective E‐Field modeling in 38 subjects (Caulfield et al. 2021). Our mean of 99.7% rMT diverges in direction from both prior estimates suggesting a greater range of variability than previously thought. Differences across studies likely reflect a combination of factors including sample age composition, DLPFC targeting approach, or perhaps diagnosis‐specific differences in required intensity. More specifically, Nahas and colleagues described an elderly sample, where greater skin‐to‐cortex distance may systematically increase intensity requirements relative to younger populations, whereas Caulfield and colleagues' analysis was conducted on patients with tobacco use disorder. Population‐level differences in DLPFC‐to‐M1 skin‐to‐cortex distance ratios have also been documented across age and sex groups, and should be considered when comparing intensity requirements across studies (Van Hoornweder et al. 2024). Together, these add to the growing evidence that a fixed‐percentage approach is inadequate for precision of dosing intensity as we stimulate outside the motor cortex.

Manifestly, precision only matters if it affects outcomes. Our ultimate question is whether TMS intensity is associated with clinical outcomes. To this end, we tested three paradigms: (1) Is closest to 120% of rMT the best? (2) Is closest to the E‐Field (V/m) needed for M1 neuronal activation (i.e., motor threshold) best? (3) Or is the highest absolute intensity as per E‐Field modeling best? Delivered %rMT was not associated with reduction in QIDS‐SR16 scores (*ρ* = −0.15, *p* = 0.436). Given the 3‐fold range of variability, it is not surprising that there is no correlation with response. When greater precision is used, both the ratio of DLPFC to M1 E‐Field strength (*ρ* = −0.49, *p* = 0.008) and absolute DLPFC E‐Field strength (*ρ* = −0.49, *p* = 0.008, 95% CI [−0.20, −0.69]) were significantly associated with depression outcomes after adjusting for d‐cycloserine group assignment. Neither sex, age, baseline severity or drug condition were significant moderators of the intensity‐outcome relationship. This negative correlation with response is contrary to the prevailing notion that higher intensity is better, although this notion also lacks clear supportive evidence. Our findings must be considered preliminary due to the exploratory design of the study, the retrospective nature of analyses, and the modest sample size.

Current clinical practice uses 120% rMT for DLPFC stimulation, a convention established by pivotal trials. However, the rationale for this specific percentage was not arrived at systematically. Early TMS trials for depression used 80% of the active motor threshold (aMT) for safety reasons (George et al. 1995), with eventual escalation to 120% rMT in the Neuronetics pivotal trial (O'Reardon et al. 2007), which may serve to account for greater coil‐to‐cortex distance at DLPFC or a “more‐is‐better” approach. Our inverse intensity‐outcome results also align with plasticity studies demonstrating optimal theta‐burst effects at subthreshold intensities (Huang et al. 2005; Chung et al. 2018; Nettekoven et al. 2014), including the original iTBS protocol, which induced robust plasticity at 80% aMT (Huang et al. 2005). Clinically, d‐cycloserine augmented trials also produced robust results at 80% rMT (Cole, Sohn, et al. 2022; McGirr et al. 2025), and similar naturalistic findings have been described (Lee et al. 2021). A common clinical concern is whether patients who are “under stimulated” (do not reach 120% rMT) can achieve full therapeutic benefit. The significant negative correlation between DLPFC/M1 E‐Field ratio and QIDS‐SR16 score change (*ρ* = −0.49, *p* = 0.008, Figure 5B) speaks directly to this question. Meaningful clinical improvement was observed across a range of delivered intensities, including subjects who received as little as ~70% of the M1 E‐Field, with no clear lower threshold where treatment becomes ineffective. These findings raise questions about the clinical assumption that reaching 120% rMT is necessary for the full therapeutic efficacy (George et al. 2010; Lee et al. 2021).

There are several limitations to our data that must be addressed before clinical implementation. Foremost among these is that while our E‐Field dosing was prospective, our comparative analyses were retrospective, without randomization or an intensity/dosing control. Our analyses are not sufficient for clinical implementation but may justify a randomized controlled trial of dosing protocols, comparing the conventional 120% of rMT to personalized precision dosing using E‐Field modeling, ideally at more than one intensity (e.g., 120% of M1‐E‐Field, 100%, and 80%). Next, it should be remembered that we used a model, and there is no objective measurement of induced electrical field in non‐invasive brain stimulation, and thus, we are comparing the relative accuracy of 120% vs. real‐time spherical models to an FEM model (SimNIBS), which is unlikely to be perfectly accurate. E‐Field extraction parameters—including the cortical coordinate of extraction, ROI sphere size, and summary metric—remain without consensus in the field, and different choices can yield meaningfully different values from the same underlying model (Van Hoornweder et al. 2023). In this study, the DLPFC target was defined anatomically (Mylius et al. 2013), rather than via functional connectivity, which may not optimally capture individual variability in prefrontal organization. Additionally, the neuronavigation system localizes the M1 by projecting the coil placement to the spherical gray matter surface, rather than directly identifying the cortical response origin, which may introduce a degree of spatial imprecision relative to approaches that utilize cortical E‐Field patterns for localization. Clinically, our single‐day accelerated protocol (1800 pulses x 10 sessions) delivers fewer total sessions than standard daily (although similar total pulse number) and accelerated clinical courses, potentially constraining the range of clinical responses and magnitude of correlation observed (Cole, Phillips, et al. 2022; Vaughn et al. 2025). Before clinical implementation of lower precision‐based stimulation, prospective testing, such as 80%, 100%, and 120% of E‐Field dose is needed.

These findings nonetheless demonstrate substantial interindividual variability (CV = 19.9%) and preliminary clinical correlations suggest that prospective trials testing different intensity levels including use of E‐Field modeling hold promise to improve clinical outcomes with TMS.

## Author Contributions

**Prem Ganesh:** writing – original draft, visualization, project administration, investigation, formal analysis, data curation. **Hakjoo Kim:** writing – review and editing, formal analysis, data curation. **Jamie Kweon:** writing – review and editing, project administration, Investigation. **Mark A. Halko:** writing – review and editing, conceptualization, supervision. **Kevin A. Caulfield:** writing – review and editing, conceptualization, supervision. **Joshua C. Brown:** writing – review and editing, conceptualization, funding acquisition, methodology, supervision.

## Funding

This work was primarily supported by the Brain and Behavior Research Foundation Young Investigator Grant (Grant No. 31748), the Cindy & Paul Gamble Fund, the Marlene Zuckerman Fund, the McLean Hospital Center of Excellence in Depression and Anxiety Disorders, the National Institutes of Health (NIH; Grant No. R01MH1389697), and the Department of Defense Advanced Research Projects Agency (Grant No. HR00112320037).

## Supporting information


**Figure S1:** Comparison of E‐Field magnitude across commonly used extraction metrics at M1 and DLPFC.
**Figure S2:** Comparison of ROI‐based E‐Field extraction across sphere sizes at M1 and DLPFC.
**Figure S3:** Skin‐to‐cortex distance at M1 and DLPFC.
**Table S1:** Subject‐level E‐Field metrics at M1 and DLPFC.
**Table S2:** QIDS‐SR16 scores for all subjects.
**Figure S4:** Clinical outcome correlations using 99th percentile E‐Field magnitude (P99).
**Figure S5:** ΔQIDS‐SR16 by DLPFC E‐Field intensity quartile.

## Data Availability

The data that support the findings of this study are available on request from the corresponding author. The data are not publicly available due to privacy or ethical restrictions.
