# Supplementary material for: Depression Improvement Correlates With Lower TMS Intensity in a Randomized Trial With Real‐Time E‐Field Modeling
Source: Hum Brain Mapp. 2026 Aug 4;47(11):e70607. doi: 10.1002/hbm.70607 (PMC13437422; doi:10.1002/hbm.70607)
Supplement: Supplementary file 1 [file HBM-47-e70607-s001.docx]

**SUPPLEMENTAL MATERIAL**

**Supplemental Figure 1. Comparison of E-Field magnitude across commonly used extraction metrics at M1 and DLPFC.** E-Field strength at the M1 hotspot (left) and DLPFC target (right) as estimated by the Nexstim real-time spherical head model and three SimNIBS FEM-derived metrics: volume-weighted ROI mean, volume-weighted ROI 99th percentile (P99), and volume-weighted whole-brain P99. Individual subjects are shown as connected dots with group boxplots overlaid. At M1, mean estimates were: Nexstim = 67.8 ± 2.3 V/m, ROI mean = 64.8 ± 1.8 V/m, ROI P99 = 126.7 ± 3.6 V/m, whole-brain P99 = 58.1 ± 1.8 V/m. At DLPFC: Nexstim = 67.8 ± 2.3 V/m, ROI mean = 67.1 ± 3.0 V/m, ROI P99 = 125.3 ± 5.5 V/m, whole-brain P99 = 58.2 ± 2.2 V/m. All values are mean ± SEM.

**Supplemental Figure 2. Comparison of ROI-based E-Field extraction across sphere sizes at M1 and DLPFC.** Volume-weighted mean E-Field magnitude within spherical ROIs of 5, 10, and 15mm radius centered at the M1 hotspot (left) and DLPFC target (right). Individual subjects are shown as connected dots with group boxplots overlaid. At M1: 5mm = 60.8 ± 2.7 V/m, 10mm = 61.6 ± 1.6 V/m, 15mm = 56.1 ± 1.3 V/m. At DLPFC: 5mm = 63.0 ± 3.0 V/m, 10mm = 62.0 ± 2.8 V/m, 15mm = 56.8 ± 2.3 V/m. Results were similar across 5 and 10mm radii at both targets, with a modest reduction at 15mm consistent with inclusion of tissue outside the focal stimulation volume. All values are mean ± SEM. The primary analysis used a 10mm radius.

**Supplemental Figure 3.** **Skin-to-cortex distance at M1 and DLPFC.** Distance from the scalp surface node nearest to the TMS coil target to the closest pial surface node, extracted from subject-specific SimNIBS tetrahedral head meshes (triangle regions 1005 and 1002, respectively) for M1 and DLPFC targets. Individual subjects are shown as connected dots with group mean ± SEM overlaid. M1: 12.7 ± 0.3 mm (range 9.9–16.9 mm); DLPFC: 11.3 ± 0.4 mm (range 7.4–14.0 mm). Skin-to-cortex distances were significantly greater at DLPFC compared to M1 (t(27) = 3.82, p < 0.001). Values are mean ± SEM.

|  | **M1** | | | | **DLPFC** | | | |
| --- | --- | --- | --- | --- | --- | --- | --- | --- |
| **Subject ID** | Mean E-Field (V/m) | Max E-Field (V/m) | 99th Percentile (V/m) | # of Tetrahedra in ROI | Mean E-Field (V/m) | Max E-Field (V/m) | 99th Percentile (V/m) | # of Tetrahedra |
| **M02** | 64.9 | 145.8 | 131.0 | 4028 | 51.9 | 118.0 | 105.7 | 6078 |
| **M03** | 51.4 | 112.3 | 100.8 | 3696 | 57.6 | 121.8 | 105.7 | 5079 |
| **M04** | 58.8 | 125.5 | 114.6 | 2708 | 55.3 | 111.5 | 98.0 | 4353 |
| **M05** | 72.6 | 151.5 | 125.7 | 5412 | 103.2 | 238.3 | 211.6 | 5479 |
| **M06** | 72.9 | 149.6 | 132.9 | 3814 | 62.4 | 145.4 | 122 | 5693 |
| **M07** | 62.4 | 138.6 | 130.1 | 2505 | 78.3 | 161.0 | 138.6 | 4572 |
| **M08** | 52.6 | 114.2 | 95.0 | 3375 | 47.5 | 93.6 | 82.1 | 4330 |
| **M09** | 62.0 | 179.5 | 122.6 | 3615 | 75.8 | 186.3 | 152.2 | 3779 |
| **M10** | 68.9 | 136.8 | 122.6 | 3348 | 83.2 | 169.0 | 151.1 | 5224 |
| **M11** | 56.6 | 144.6 | 113.5 | 4178 | 56.4 | 156.0 | 123.1 | 4808 |
| **M12** | 57.4 | 139.1 | 114.9 | 3715 | 70.2 | 161.7 | 131.0 | 4396 |
| **M13** | 63.7 | 123.2 | 112.9 | 4819 | 71.8 | 130.3 | 122.7 | 3238 |
| **M14** | 81.9 | 179.2 | 156.1 | 2855 | 67.6 | 150.5 | 134.8 | 4311 |
| **M15** | 63.0 | 150.8 | 121.8 | 3347 | 64.8 | 127.5 | 112.0 | 4173 |
| **M16** | 63.5 | 144.7 | 128.5 | 3497 | 71.5 | 131.8 | 121.9 | 4613 |
| **M18** | 56.8 | 161.1 | 139.6 | 4743 | 53.3 | 123.0 | 115.9 | 3975 |
| **M19** | 66.1 | 119.0 | 109.7 | 2316 | 79.3 | 153.0 | 142.2 | 4590 |
| **M20** | 60.3 | 225.0 | 129.4 | 2836 | 64.4 | 142.8 | 112.8 | 3817 |
| **M21** | 53.9 | 148.4 | 115.4 | 3075 | 46.3 | 104.8 | 84.4 | 3643 |
| **M22** | 61.0 | 138.3 | 129.9 | 3015 | 58.6 | 169.9 | 117.7 | 4021 |
| **M23** | 91.1 | 183.6 | 169.7 | 2939 | 100.8 | 245.0 | 188.1 | 4035 |
| **M24** | 61.2 | 116.9 | 106.0 | 2387 | 65.0 | 141.7 | 109.3 | 4816 |
| **M25** | 74.7 | 151.0 | 135.2 | 3633 | 66.3 | 137.7 | 127.5 | 3888 |
| **M26** | 66.2 | 199.5 | 146.5 | 3443 | 37.5 | 97.0 | 89.5 | 3265 |
| **M27** | 49.9 | 113.3 | 92.9 | 4149 | 64.3 | 119 | 106.4 | 4476 |
| **M28** | 72.0 | 177.9 | 155.1 | 4612 | 73.3 | 149.7 | 128.5 | 4165 |
| **M29** | 69.7 | 206.0 | 148.3 | 4117 | 54.6 | 132.1 | 109.9 | 4687 |
| **M30** | 78.2 | 209.5 | 148.2 | 3534 | 98.1 | 190.7 | 164.0 | 2580 |
| Mean | 64.8 | 153.0 | 126.7 | 3561.1 | 67.1 | 146.8 | 125.3 | 4360 |

**Supplemental Table 1. Subject-level E-Field metrics at M1 and DLPFC.** Volume-weighted mean, maximum, and volume-weighted 99th percentile E-Field magnitude within the 10mm spherical ROI, and number of gray matter tetrahedra within the ROI, for each subject at the M1 hotspot and DLPFC target. All E-Field values are in V/m and were extracted from subject-specific SimNIBS tetrahedral head models. Note that tetrahedra are irregular mesh elements and are not equivalent to voxels from volumetric imaging.

|  | **QIDS-SR16 Scores** | | |
| --- | --- | --- | --- |
| **Subject ID** | Baseline | 1-Week Post-TMS | $\Delta$ (Pre – Post) |
| **M02** | 15 | 4 | 11 |
| **M03** | 8 | 11 | -3 |
| **M04** | 25 | 21 | 4 |
| **M05** | 13 | 13 | 0 |
| **M06** | 17 | 9 | 8 |
| **M07** | 12 | 8 | 4 |
| **M08** | 13 | 6 | 7 |
| **M09** | 21 | 17 | 4 |
| **M10** | 9 | 11 | -2 |
| **M11** | 11 | 2 | 9 |
| **M12** | 22 | 22 | 0 |
| **M13** | 18 | 20 | -2 |
| **M14** | 8 | 8 | 0 |
| **M15** | 11 | 11 | 0 |
| **M16** | 7 | 11 | -4 |
| **M18** | 17 | 14 | 3 |
| **M19** | 14 | 12 | 2 |
| **M20** | 10 | 5 | 5 |
| **M21** | 13 | 9 | 4 |
| **M22** | 12 | 6 | 6 |
| **M23** | 17 | 16 | 1 |
| **M24** | 8 | 12 | -4 |
| **M25** | 9 | 5 | 4 |
| **M26** | 14 | 10 | 4 |
| **M27** | 21 | 17 | 4 |
| **M28** | 20 | 19 | 1 |
| **M29** | 14 | 9 | 5 |
| **M30** | 16 | 15 | 1 |

**Supplemental Table 2. QIDS-SR16 scores for all subjects.** QIDS-SR16 was collected at baseline on the treatment day (Pre) and 1-week after TMS treatment (Post). Score change was calculated as Pre – post, where positive values indicate an improvement in depressive symptoms.

**Supplemental Figure 4. Clinical outcome correlations using 99th percentile E-Field magnitude (P99).** Correlations between reduction in QIDS-SR16 score and three dosing frameworks using volume-weighted 99th percentile E-Field as the primary metric: (A) delivered %rMT, (B) DLPFC E-Field as a percentage of M1 E-Field, and (C) absolute DLPFC P99 E-Field magnitude. Placebo and D-cycloserine group assignments are indicated by color. Regression lines with 95% confidence intervals are shown. Unadjusted and group-adjusted Spearman correlations are reported. **A**: ρ = -0.15, p = .436 (adj: ρ = -0.16, p = .426). **B**: ρ = -0.41, p = .032 (adj: ρ = -0.40, p = .033). **C**: ρ = -0.34, p = .076 (adj: ρ = -0.40, p = .035, 95% CI [-0.01, 0.61]). Results were directionally consistent with the primary mean E-Field analyses. ΔQIDS-SR16 = pre minus post; positive values indicate improvement.

**Supplemental Figure 5. ΔQIDS-SR16 by DLPFC E-Field intensity quartile.** Change in QIDS-SR16 score (pre minus post; positive values indicate improvement) across four equally sized quartiles of mean DLPFC E-Field strength (n = 7 per quartile). Quartile boundaries are shown on the x-axis in V/m. Individual subjects are shown as scatter points overlaid on boxplots. Q1 (lowest): 5.4 ± 2.6 points (median = 4.0); Q2: 3.3 ± 4.7 points (median = 4.0); Q3: 0.4 ± 3.8 points (median = 0.0); Q4 (highest): 1.4 ± 2.0 points (median = 1.0). Kruskal-Wallis H(3) = 8.21, p = .042. Values are mean ± SD.
